# Childbearing through the Lens of Women with Minor Thalassemia: A Qualitative Study

**DOI:** 10.31661/gmj.v8i0.1429

**Published:** 2019-08-17

**Authors:** Khadijeh Sarayloo, Zahra Behboodi Moghadam, Khadijeh Mirzaii Najmabadi, Sharon Elizabeth Millen, Mohsen Saffari

**Affiliations:** ^1^Nursing and Midwifery Schools, Mashhad University of Medical Sciences, Mashhad, Iran; ^2^Reproductive Health Department, School of Nursing and Midwifery, Tehran University of Medical Sciences, Tehran, Iran; ^3^New Baby Programme, Centre for Evidence & Social Innovation (CESI), School of Social Sciences, Education and Social Work, Queen’s University Belfast, United Kingdom; ^4^Health Research Center, Life Style Institute, Baqiyatallah University of Medical Sciences Tehran, Iran; ^5^Health Education Department, School of Health, Baqiyatallah University of Medical Sciences, Tehran, Iran

**Keywords:** Thalassemia, Pregnancy, Qualitative Research, Iran

## Abstract

**Background:**

It is essential to provide key information and support to every woman regarding pregnancy and childbirth to enable all women to make a fully informed decision regarding their choice to reproduce. Of particular importance; however, is that women who suffer from a specific condition that increases risks associated with reproduction, to receive specific information regarding such risks and health complications regarding pregnancy and childbirth. This study aims to explore the feelings, experiences, and needs of women with minor thalassemia in relation to childbearing.

**Materials and Methods:**

This sample comprised of 12 Iranian women with minor thalassemia who attended to local health centers in Minudasht, Golestan province, from October 2017 to January 2018. The purposeful sampling technique was employed. The data were collected through deep semi-structured interviews, analyzed using conventional content analysis and processed by using the Graneheim and Lundman method.

**Results:**

The findings were based on the participants’ experiences of pregnancy and fertility. Three major themes emerged; ‘Emotional and Physical Experience,’ ‘Satisfaction’ and ‘Social and Cultural Issues.’ The most common problem identified was the impact of emotional problems and difficulties regarding the negative implications of thalassemia on reproduction as well as the views and perceptions of family members and those within the community.

**Conclusion:**

From the findings, it is evident that thalassemia imposes a heavy psychosocial burden on those women who suffer from the condition. Local health centers in areas most affected by thalassemia would be considered the most suitable venues to introduce key health educational interventions.

## Introduction

Thalassemia is the most common hereditary disease in the world. Approximately 6% of the global populations are carriers of the β-thalassemia mutant gene [1]. The areas of highest prevalence are in North and South Iran, particularly within the Golestan and Mazandaran provinces where prevalence rates of those affected are between 10% to 13%[2]. Thalassemia poses a remarkable impact on women’s lives in physical, psychological, and social aspects [3]. The added stress of pregnancy, for example, can cause a deterioration of maternal status [4] exposure to subfertility and frequent abortions, [5, 6] a demand for ovulation stimulation and intrauterine fertilization [7]. Other related consequences include unwanted pregnancies, increased risk of maternal and fetal complications, abnormal pregnancy outcomes, preterm labor and a higher risk of requiring a cesarean section [6, 8, 9]. Cultural and social factors, in general, can have a large impact on the value ​​and belief system of those within certain communities, in terms of health, life, happiness, family continuity and race [10, 11], including views and expectations regarding childbearing [12]. The cultural and societal value and expectation placed upon childbearing, not only can it largely motivate women to reproduce but often dictates the number of babies she is expected to produce [13-15]. Those women with minor thalassemia are often afraid and apprehensive about the prospect of falling pregnant because of the increased likelihood of their child having major thalassemia. Despite this fear; however, those affected are often impelled to child bear because of the strong influence of their cultural expectations and views [16-19]. It is therefore imperative to equip women with relevant information via targeted reproductive health education-focused interventions so as they can, in turn, develop and thus demonstrate more autonomy over their own informed decision-making regarding their desire to fall pregnant, while considering all potential health risks and complications involved. However, for achieving this aim, the active engagement and participation of those affected are essential. Therefore, these women must be encouraged to attend targeted health education interventions by placing more value on the importance of healthcare rather than social and cultural expectations. Potential methods of achieving this would be to provide key information leaflets to all those affected via General physicians at the health centers or to distribute a community-wide mail drop of information leaflets. Previous qualitative research to date in relation to thalassemia has focused largely on the severe biological, and social impact of childbearing on women affected, including infertility, prenatal diagnosis and/ or pre-term labor. However, in recent years, there has been a growing interest specifically on childbearing and the impact of thalassemia [15, 20-22]. In general, women experience various degrees of acceptance and attachment in relation to their unborn child and can perceive and express their pregnancy experience in several contrasting ways, largely dependent upon specific circumstances regarding the pregnancy. These circumstances are largely dependent upon a wide range of social contexts, which can differ significantly in terms of material, cultural resources and constraints [23, 24]. The autonomy of women as to whether they make an informed decision to fall pregnant, as well as their motivation for childbearing, is different in every context [16, 20]. The way in which women with minor thalassemia experience childbearing has not been investigated to date in Iran. Therefore, it is crucial to explicitly establish the feelings and perception of those women affected in terms of their fears, concerns and perceived level of support required regarding the experience of pregnancy and childbearing within a safe neutral environment. This study aims to contribute to this significant gap in the literature by exploring the feelings, experiences, and needs of women with minor thalassemia in relation to childbearing in Iran.

## Materials and Methods

A qualitative study was conducted using a deep semi-structured interview approach to explore in depth, the women’s’ experience of childbearing. The data was obtained objectively from participants without the use of leading questions or personal bias. A conventional content analysis approach was employed. The research questions were as follows:

1- What is the experience of childbearing and pregnancy for women affected by minor thalassemia?

2- What are the views of women affected by thalassemia regarding pregnancy and childbearing?

### Setting

The study was carried out at Minudasht health center in Golestan province, in Iran.

### Participants

Women with minor thalassemia (whose husbands also had the condition) were selected by using purposeful sampling. All the women were of Iranian origin. Women were invited to participate in a face-to-face interview regarding their views, experience of pregnancy and childbearing. In total, 12 women agreed to be contacted again by the researcher with the view of consenting to take part in the study. Data saturation was reached by the 12 interviews, and thus recruitment ended at that point. The study’s inclusion criteria were as follows: Married women with minor thalassemia (whose husbands also had the condition), aged between 15 to 49 years old, patients under the supervision of Minudasht health center, had a history of at least one pregnancy, consented to participate in the research. Notably, five women out of the sample of 12 (42%) had already undergone an abortion due to the unborn child having major thalassemia.

### Data Collection

Recruitment and data collection took place between October 2017 and January 2018 after permission from the Ethics Committee of Mashhad University of Medical Sciences was granted (ethical code: IR.MUMS.REC.1396.6). The purposeful sampling technique employed aimed to capture a significant contrast in terms of variations in experience. All participants were aware of the purpose and method of the study, and written consent was obtained from each participant prior to the interview. The interviews were conducted by the first author and recorded with the permission of the participants and took place in a location of the woman’s choice (most often in a private room in the health center) and lasted between 35-50 minutes. At the beginning of the interview, each participant was asked a series of demographic questions (Tabe-1). The interview then continued with a broad in-depth question relating to the woman’s individual experience of pregnancy/pregnancies and, thereafter, a semi-structured schedule was employed to address further questions. Prompts were provided when required to ensure that all topics were discussed. A sample of the interview questions are in Table-1. In all interviews, participants were encouraged to speak freely about their experiences of pregnancy. All the interview recordings were transcribed verbatim and then encoded initially by the first author upon completion. After analysis of each interview, a subsequent interview was conducted with the next participant.

### Data Analysis

The data were analyzed using the conventional content analysis approach and processed using the Graneheim and Lundman method [25] as follows:

1- First, the interviews were conducted at the earliest opportunity after recruitment and consent obtained. After 12 interviews, complete data saturation was achieved. Upon the completion of each interview, the recording was listened to several times to ensure that the interview content was accurately transcribed.

2- The data was then analyzed

3- The text of the interviews were divided into several meaningful units and then coded.

4- The units of meaning were summarized and derived in code form.

5- The codes were grouped based on similarities and differences under the same themes and sub-categories.

6- Coding and analysis were completed by hand over several months. Initial coding schemes were developed based on the interview guides and existing literature and were subsequently adapted and updated upon the completion of successive interviews and coding process. Four members of the research team independently double-coded several transcripts and then met to resolve coding differences through discussion, agreement, and development of new codes. MAXQDA10 was used to organize the data, code transcripts, and generate reports. For this analysis, we focused on each individual’s experiences and views of childbearing.

### Trustworthiness

Every attempt was made to capture the essence of the phenomenon for the participants as a whole while staying close to individuals’ experiences. After each interview, the interviewers recorded their impressions and reactions of the participant. Researcher memos were later reviewed and compared with the data. An audit trail, with regards to the protocol and records kept regarding any deviation from such, was implemented at the beginning of the research project [26]. To ensure the accuracy of the data, the proposed criteria of Lincoln and Guba were applied [27]. Lincoln and Guba (1985) suggested that the value of a qualitative research study is strengthened by its trustworthiness. Trustworthiness involves establishing credibilityor confidence in the “truth” of the findings; transferabilityor ability to apply the results to other contexts; dependabilityor showing consistent findings that can be repeated and; confirmabilityor the extent to which the findings are shaped by the participants views and not skewed by researcher bias, motivation, or interest. Credibility was confirmed by studying the participants’ and researchers’ reviews, asking a number of participants to review the interview transcripts and the derived codes to confirm as to whether they were an accurate representation of their experiences. Data transferability was confirmed by explicitly providing detailed information regarding participants’ characteristics as well as the method of data collection and analysis, while also providing examples of the participant interview questions to allow others to replicate the research [27].

## Results

### 1. Demographic Characteristics

The mean age of the 12 participants interviewed was 35 years (ranged 20–45 years). Five women reported that they already had one abortion because of major thalassemia. Of the 12 participants, three women worked as midwives while eight were housewives. One participant was illiterate. The demographic characteristics of the study’s participants are listed in Table-2. Three major themes were extracted from the data ( Table-3). These themes included: ‘Emotional and Physical Experience,’ ‘Satisfaction’ and ‘Social and Cultural Issues.’

### 2. Emotional and Physical Experience

The most common response provided by participants regarding their childbearing and pregnancy experience was that of wanting to have someone to love them. Data related to this theme were identified by further questioning the participants to discuss their experiences of undergoing pregnancy with thalassemia. This theme included two sub-categories; negative psychological feelings and physical problems faced by women with minor thalassemia.

#### 2.1. Negative Psychological Feelings and Physical Problems

Several women emphasized concerns of having to endure specific physical problems associated with their pregnancy. For example, one stated:*“When I was pregnant, it was so hard for me. It was very difficult to test. After testing, I had to rest at home for a week; I felt a lot of pain and stress; I was in bed because of fearing the prospect of abortion. During the test, I was worried about touching the needle in the baby”* (Interviewee Code 3).One of the participants who had to abort her pregnancy because of a prenatal diagnosis of major thalassemia described her experience of pregnancy as follows: *“It was very hard to me. I had fulltime stress about health of my baby. When I became pregnant, I had to be tested. I realized that my baby had major thalassemia and so I had to abort it. My husband was upset and left the clinic, but I had to wait and go for a personal consultation. I was disappointed and had to abort my baby... It was so hard for me, both physically and mentally…”* (Interviewee Code 8). Others reported that they had experienced physical complications during pregnancy and required special care. One participant for example, who was a midwife, expressed her feeling about her pregnancy as follows: *“During pregnancy because of problems, such as dizziness and anemia, my general wellbeing was affected. I was not prepared and did not take folic acid. From this point of view, it was a bit hard to accept my pregnancy. After I accepted my pregnancy and continued it, I had nausea and dizziness in the first four months. I wasn’t happy to experience it…”* (Interviewee Code 11).

### 3. Satisfaction

Satisfactionemerged as another important main theme. Mothers discussed the feelings and emotions relating to their experience of childbearing. To also ascertain the general impact of thalassemia on childbearing, women were asked to talk about their feelings regarding women with minor thalassemia becoming pregnant. This theme includes the following three sub-categories: the feeling of value, the stability of the family and spiritual support.

#### 3.1. The Feeling of Value

In general, the participants reported that they enjoyed their pregnancies without thinking about thalassemia. For instance, one participant said: *“I had a great feeling of pride, when I realized I was pregnant”* (Interviewee Code 8).

Some women felt elated at the prospect of the motherhood experience

One participant stated:* “I had a great feeling regarding motherhood. It was a great energy, like my life was warming up. If you have no baby, you have no home… A baby makes life better, and you’re going to progress”* (Interviewee Code 8).

#### 3.2. Stability of Family

The next sub-category was the stability of the family, which reflected the family’s response and support provided regarding the woman’s pregnancy. One of the participants stated: *“A baby plays a very important role in the relationship between the husband and wife. When my child was born, my marital relationship improved and became more intimate, and my marital life became stronger than before the arrival of my child”* (Interviewee Code 6).

#### 3.3. Spiritual Support

Spiritual support (which includes beliefs and other spiritual and religious issues) was identified as another sub-category of ‘Satisfaction’ - this was one of the most important aspects of these women’s responses to being pregnant. Women expressed that their belief in God helped them to better cope during pregnancy. One woman stated: *“I had no idea. I believe in God. When I found out I was pregnant, the doctor asked me to have an abortion because of thalassemia, I was so upset and prayed to God a lot. My husband wouldn’t agree to the abortion. We were put under the supervision of a doctor, and he advised us to take a medication before prenatal testing. I kept praying for a healthy baby”* (Interviewee Code 2).

### 4. Social and Cultural Issues

Social and cultural issues emerged as a third main theme as it was regarded by participants as a significant factor that greatly influenced the suffering of women with thalassemia. It comprised of two sub-categories, which separated social issues from cultural issues. An important factor that influenced these women’s experiences was the reaction of the community and family. This had a great psychological effect on their perception of their pregnancy.

#### 4.1. Social Issues

Acceptance of the condition by others within the community as well as having to deal with those women without the condition who experienced normal pregnancies were important factors. One participant described her friends and neighbors’ view of her pregnancy as: *“People say that when women with thalassemia become pregnant, they pray and hope to have healthy babies. My friends said to me: why did you become pregnant again? You’re sick, and your baby won’t be healthy. I became upset and distracted. The community’s view was not good”* (Interviewee Code 8). The views of some participants regarding social factors were as follows: *“People have a different opinion. They think we should not have baby. People have a low level of literacy; they know very little about thalassemia and have a negative attitude toward this disease”* (Interviewee Code 5).

#### 4.2. Cultural Issues

Cultural issues are important factors that affected the participants. One woman reported:

*“Perception of people about us isn’t good. They think that we shouldn’t become pregnant because of thalassemia. They believe that the child will be ill, so we do not have the right to become pregnant since we would spend a lot financially if we got pregnant. There is no culture…”* (Interviewee Code 6).

## Discussion

To our knowledge, this is the first study in Iran which has explored the individual views and experiences of mothers with minor thalassemia regarding childbearing. Because of its exploratory and qualitative design, the present study has contributed to the identification of significant issues relating to the experience and views of women with minor thalassemia regarding their childbearing and pregnancy experience. The study employed a qualitative approach to obtain rich, in-depth data, particularly from an exploratory perspective of the mothers’ experience and views. There have been several studies conducted using a range of methodologies and samples regarding views and experiences of motherhood and childbearing.

### Emotions and Physical Experience

Aujulat *et al*. (2010), for instance, conducted a study which aimed to better understand the views of adolescent mothers regarding their experiences of pregnancy and motherhood. Several of the participants stated that they felt valued after childbearing because for them, motherhood created a sense of maturity and responsibility. The findings, in general, indicated that adolescents were delighted and proud of becoming a mother [28]. Our findings from this present study in part concur with those of Aujulat *et al*. [28] as the majority of participants were generally excited about their pregnancy and had a sense of satisfaction because of becoming pregnant, despite the increased risks associated with their health condition.

### Support

In the present study, the support category was an important aspect mentioned by the participants. The results demonstrate that thalassemia can have a substantial effect on the emotional and social lives of those affected. In general, participants perceived thalassemia as a distressing condition that negatively impacted their childbearing experience and social lives. Women with thalassemia experience physical and psychological problems and often struggle to meet the needs of their child without the support of others. According to our findings, participants highlighted the desire and necessity for the provision of a comprehensive support system both within the home and throughout their community. Therefore, spousal, familial and societal support can have a significant positive effect on the physical and mental wellbeing of those affected. This particular aspect of the findings concurs with previous studies that have found similar concerns [29, 30]. The consensus from these previous studies was that health care interventions must provide psychological support for women with thalassemia to facilitate the use of better coping strategies in relation to their distress regarding having minor thalassemia. The provision of support for these women is imperative given the rigid views and expectations of the culture and society in which they live. Our findings also concur with a study by Abdul *et al*. [31] that investigated the impact of thalassemia upon patients and their families. The findings indicate that thalassemia can have a significant negative psychosocial impact both upon patients and family members. This again emphasizes the need for the availability of social and psychological support services. Another significant finding from this research was the women’s desire for information; they spoke extensively about their educational needs which were generally very limited, particularly in terms of the facts and knowledge regarding their condition. These findings are consistent with previous studies [32, 33] where mothers reported the need for information about the condition thalassemia, what causes it, what the health risks are and how it can be prevented. Lack of knowledge about thalassemia and its treatment has thus been demonstrated through our findings and similar studies to cause unnecessary anxiety and emotional distress among women and their families [34]. Therefore, the implementation of tailored health educational programs that include professional psychosocial support and located within the health centers is strongly recommended. The results of this study, similar to other qualitative studies, are not generalizable to all those women affected with minor thalassemia due to its exploratory nature and small sample size (n=12) all of which came from the same geographical area.

## Conclusion

There is a wealth of previous studies that have focused on thalassemia. However, most of these studies employed a quantitative design and thus did not have the rich in-depth qualitative data derived from the interview regarding women’s own views, experiences, perceptions, feelings and the importance of support. This rich data provides a much deeper understanding of the concept and the main concerns that those affected have to endure. These themes and subcategories can be used as a structure for a larger multi-site study with a sufficient sample size for the findings to be generalizable to this patient group as a whole. These findings highlight that it is of upmost importance to provide health education and support interventions including professional psychosocial support. Psychosocial support is necessary for women to accept their pregnancy, i.e., through the validation and support of family and others. The stigma associated with thalassemia is one of the main obstacles to the disclosure of having the condition resulting in failure to receive adequate care during pregnancy. Childbearing was also found to be a major feature in the status and fertility of women within Iran. Thus, a recommendation from our findings is the provision of psychologists and/ or trained counselors specific to this patient group to be available within the health education interventions to address the issue of mental health. These programmes would aim to increase people’s awareness about thalassemia and its prevention community-wide.

## Acknowledgment

This study was supported by the Vice-chancellor for Research & Technology of Mashhad University of Medical Sciences (grant number: 951364).

## Conflict of Interest

The authors report no conflicts of interest.

**Table 1:** Example of Initial Interview Questions

**Interview Questions**
**1.** Express your understanding and experience of pregnancy; **2.** What are your feelings in terms of women with minor thalassemia falling pregnant? **3.** What are the conditions of pregnancy for women with thalassemia? If you consider it to be difficult, how difficult is it? **4.** How did thalassemia affect your pregnancy?

**Table 2:** Demographic Characteristics of the Participants

**Code**	**Age**	**Education**	**Occupation**	**Spouse occupation**	**Length of marriage**	**Number of pregnancies**	**Number of children**	**Number of abortions**	**Number of children with major thalassemia**
**1**	40	Bachelor	Midwife	Employee	17	2	2		
**2**	23	Elementary	Housewife	Workman	5	1	1		
**3**	36	Diploma	Housewife	Employee	10	2	1	1	
**4**	20	Elementary	Housewife	Farmer	4	2	1	1	
**5**	40	Elementary	Housewife	Worker	24	4	4		1
**6**	36	Elementary	Housewife	Plumber	17	3	3		
**7**	26	Elementary	Housewife	Worker	7	1	1		
**8**	34	Illiterate	Housewife	Worker	7	4	4		1
**9**	31	Diploma	Housewife	Worker	9	4	2	2	
**10**	42	Elementary	Housewife	Driver	21	3	3		
**11**	45	B.A	Midwife	Teacher	20	2	2		
**12**	38	B.A	Midwife	Employee	13	1	1		

**Table 3:** Categories, Sub-Categories, and Themes for Mothers’ Pregnancy Experiences with Minor Thalassemia

**Theme**	**Sub -Category**	**Related topics**
**Emotional and physical experience**	Negative psychological feelings	Stress and anxiety
		Discomfort and being afraid that the child may carry the disease
		Regret
		Low self-confidence
		Concerned about forced abortion
		Worry
		Mental pressure
	Physical problems	Painfulness of the test
		Mother’s weakness
		Symptoms of intense nausea and dizziness
		Difficult pregnancy
		Physical difficulty related to abortion
**Satisfaction**	The feeling of value	The feeling of motherhood
		The feeling of pride
		Rising dignity
		Enjoyable pregnancy
	Stability of family	Improved marital relationship
		Collaboration and attention of the spouse
		The spouse’s feeling of responsibility owing to childbirth
		The family encouragement
Spiritual support	Resort to God
	Thank god
	Pray	
**Social and cultural issues**	Social issues	Low awareness of the community
		The family disagreement
		People’s negative view
		Stay at home all the time
	Cultural issues	Poor culture of People
		Stigma
		People’s wrong beliefs
